# Acute Vertigo After COVID-19 Vaccination: Case Series and Literature Review

**DOI:** 10.3389/fmed.2021.790931

**Published:** 2022-01-06

**Authors:** Paola Di Mauro, Ignazio La Mantia, Salvatore Cocuzza, Pasqua Irene Sciancalepore, Deborak Rasà, Antonino Maniaci, Salvatore Ferlito, Isabella Tundo, Roberta Anzivino

**Affiliations:** ^1^Department of Medical and Surgical Sciences and Advanced Technologies “G.F. Ingrassia”, ENT Section, A.O.U. Policlinico “G.Rodolico-San Marco”, University of Catania, Catania, Italy; ^2^Centre of Phoniatry and Rehabilitation of Communication Disorders - Azienda Sanitaria Locale Lecce, Lecce, Italy; ^3^Otolaryngology Unit, Di Venere Hospital, Bari, Italy

**Keywords:** HINTS examination, COVID-19, acute vertigo, dizziness, central vertigo, peripheral vertigo, tinnitus, vaccine

## Abstract

**Objective:** The aim of this study was to present some cases of acute vertigo potentially related to the coronavirus disease 2019 (COVID-19) vaccine and review the available literature about cochleovestibular dysfunction after the COVID-19 vaccination.

**Methods:** In the period from May to July 2021, we evaluated 33 patients (mean age 54.3 ± 14.1) with “acute vertigo” post COVID-19 vaccination. A detailed medical history was taken on comorbidities, types of vaccines received, and symptoms associated. All patients underwent otoneurological evaluation, such as head impulse test, nystagmus evaluation, test of skew (HINTS) examination. Head shaking test-induced nystagmus, hyperventilation-induced nystagmus, and parossistic positional nystagmus were studied to search for vestibular impairment.

**Results:** Symptoms included 16 patients (48.5%) with objective vertigo, 14 patients (42.4%) with subjective vertigo, and 3 patients (9.1%) with dizziness. Of the associated ear, nose, and throat (ENT) symptoms, the most expressed was tinnitus (18.2%). Bedside examination showed absent nystagmus in 7 patients (21.2%), 9 patients (27.3%) had horizontal or rotatory nystagmus, 17 patients (51.5%) had a vertical or oblique nystagmus, negative HST, or “central HINTS.”

**Discussion and Conclusions:** The 9 patients had an evoked nystagmus pathognomonic for benign paroxysmal positional vertigo; in the remaining 17 cases, peripheral vestibular dysfunction could be excluded and central disorder may be suggested. Due to the prevalence of nystagmus of non-peripheral origin, a central nervous system involvement could not be excluded. However, due to the small sample size, a definite cause–effect relationship between vaccination and vertigo cannot be inferred. In light of expected third dose, large-scale and well-designed studies are needed to better define possible adverse reactions of the COVID-19 vaccine.

## Introduction

Severe acute respiratory syndrome coronavirus (SARS-COV-2) infection has led to a global pandemic and a public health crisis, resulting in over 4,806,841 deaths at the time of publication (1).

The efforts of the scientific community to prevent coronavirus disease 2019 (COVID-19) associated mortality and morbidity have resulted in multiple vaccines worldwide available and approved for use.

Severe acute respiratory syndrome coronavirus spike (S) glycoprotein is the main target for current vaccines, since antibodies directed against SARSCoV-2 spike can block the fusion between the virus and host cell membrane, inhibiting the infection (2, 3).

Currently, authorized vaccines for COVID-19 include the mRNA vaccines: BNT162b2 (Pfizer/BioNTech) and mRNA-1273 (Moderna) and the adenoviral-vectored vaccines: ChAdOx1 nCoV-19 (University of Oxford/AstraZeneca) and Ad26.COV2.S (Janssen).

Pfizer/BioNTech is currently the most widely used vaccine in the Italian vaccination campaign (71%), followed by AstraZeneca (16%), Moderna (11%), and COVID-19 Janssen vaccine (2%).

Adverse effects observed in Italy after administration of these vaccines, are recorded in the COVID-19 Vaccine Surveillance Report drawn up by the Italian Medicines Agency (AIFA) (4). As of August 2021, 91,360 reports of adverse events following vaccination have been entered in the National Pharmacovigilance Network, out of 76,509,846 vaccine doses (119/100,000 administered doses). Approximately 86.1% of adverse effects reports entered refer to non-serious events, and 13.8% to serious adverse events.

The most reported adverse events fall within general diseases as fever, injection site pain, asthenia, followed by pathologies of the nervous system, such as headache and paresthesia, by pathologies of the musculoskeletal system and of the connective tissue, mostly musculoskeletal pain, and by gastrointestinal diseases, generally nausea, vomiting, and diarrhea. Rare are psychiatric disorders, cardiac, blood, and lymphatic system disorders, eye, ear, and labyrinth disorders. Very rare are anaphylactic reactions, myocarditis/pericarditis, and facial nerve paralysis. Very rare adverse events related to Astra Zeneca include acute and subacute neuropathies (such as, Guillain–Barré's syndrome) and intracranial or atypical venous thrombosis with or without thrombocytopenia.

A recent systematic review and meta-analysis of clinical trials were conducted on the incidences of nervous and muscular adverse events (NMAEs) after COVID-19 vaccination. The incidence of NMAEs was 29.2% in the vaccine group and 21.6% in the control group, in a total of 15 randomized blinded controlled clinical trials. Systemic neurological symptoms included migraine, dizziness, vertigo, and syncope (5).

Audiovestibular side effects for the COVID-19 vaccines, as already mentioned, are generally categorized as “ear and labyrinth disorders,” which include a wide range of clinical expression.

Few reports of audiovestibular symptoms after the administration of all four types of vaccine were notified by the Italian Pharmacovigilance Network (4). Recently Parrino et al. (6) published three cases of sudden unilateral tinnitus following BNT162b2 mRNA-vaccine injection, which rapidly resolved in 2 out of 3 cases.

In addition, Tseng et al. reported a single case presenting with sudden-onset tinnitus and cochleopathy after his first dosage of COVID-19 vaccine, reversible and recoverable under conservative steroid management (7).

Besides, the US Vaccine Adverse Event Reporting System (VAERS) database (8) cites possible adverse reactions involving the cochleovestibular system: 12,787 reports of tinnitus among 1,302,332 COVID-19 vaccine total adverse events, 1,627 reports of hypoacusis, 8,504 reports of vertigo, 254 of positional vertigo, and 133 of vestibular neuronitis.

It is worth noting that acute vertigo syndrome could represent an overlap between ear/labyrinth and nervous system disorders, especially if nystagmus presence/absence or peripheral/central etiopathogenesis have not been investigated.

In this work, we present some cases of acute vertigo potentially related to the vaccine, to enlarge the available literature and, if possible, suggest hypotheses about the origin of vestibular dysfunction after the COVID-19 vaccination.

## Materials and Methods

### Participants

During the period from May 1 to July 30, 2021, in this observational retrospective study, we evaluated 33 patients (7 men and 26 women; mean age 54.53 ± 14 years) with “acute vertigo” after COVID-19 vaccination. These patients arrived at the vestibular clinic from the Emergency Room of our Hospital or after the request of a primary care physician. The patients reported vertigo or dizziness not more than 48 h after the COVID-19 vaccination. No patient had the COVID-19 disease before administering the vaccine. Inclusion criteria: all adult subjects (>18 years old) referred for acute vertigo after the COVID-19 vaccination were enrolled. Exclusion criteria: subjects with acute vertigo onset before COVID-19 vaccination.

For all patients, we performed: bedside examination with vestibulospinal stability tests, head impulse test, nystagmus direction, testing skew (HINTS) examination, head shaking test (HST), hyperventilation-induced nystagmus (HIN), and positional nystagmus maneuvers.

The Research Ethics Committee of Catania 1, G Rodolico-San Marco University Hospital, approved the study protocol (Permit Number: 242/2021/PO). The study was conducted in accordance with the Declaration of Helsinki and all participants provided their written consent.

### Bedside Examination

The bedside examination was performed at the moment of the hospital admission. First of all, a complete medical history was taken: past and proximate medical history, paying particular attention to comorbidities and cardiovascular risk. In the proximate medical history, we asked for objective or subjective vertigo and dizziness, vertigo/dizziness length, if the patient had neurovegetative symptoms, for trigger of vertigo, visual impairment, tinnitus or hearing loss onset, or other symptoms associated with acute vertigo.

After medical history acquisition, we evaluated equilibrium of a patient with vestibulospinal stability tests:

- Romberg test, having the patient stand in tandem or on one foot with eyes open and closed;
- Fukuda stepping test, performed by marching in place with eyes closed for 30 s and noting any excessive turning suggestive of a vestibular imbalance.
- Finger-nose-finger, heel-knee-shin, rapid alternating movements, to evaluate cerebellar function and search potential dysmetria and/or adiadochokinesia.

### HINTS Examination

Head impulse test, nystagmus direction, testing skew (HINTS) examination is a triad component that we routinely perform in our clinic, and it consists of three steps: head impulse test (HI), nystagmus direction (N), testing skew (TS). HINTS was developed as a test to assess patients with acute vestibular syndrome (AVS), defined like the acute onset of vertigo, dizziness, gait instability, presence of neurovegetative symptomatology (nausea and vomiting), head movements intolerance, and presence of nystagmus (9–12).

Head impulse testing is used in both unilateral and bilateral vestibulopathy. It is to remember that a normal response to a rapid and passive eye movement during a fixation on central target (in this case, usually the nose of examiner) is an equal and opposite eye movement of the same magnitude. Moving the head of a patient toward or away from center position, vestibulo-ocular reflex (VOR) does not change; instead, if there is a peripheral vestibular damage, VOR is damaged and the acceleration signal to move eyes is impaired and resulting in gain loss. HIT is considered “positive” (or abnormal) when rapid movements of a patient's head bring to a fixation loss of the eyes and a corresponding refixation saccade: this is common in people with peripheral vertigo (for instance, in vestibular neuritis). Instead, central vertigo has a “negative” (or normal) HIT, and this is because the VOR is not damaged and the eye of a patient remains fixed on target (12).

Nystagmus direction analysis is very important to differentiate a central from a peripheral vertigo: pseudo-spontaneous nystagmus, gaze induced, direction changing nystagmus, head shaking nystagmus, pure torsional, or pure vertical nystagmus in patients with AVS are signs of possible central lesion. Instead, a spontaneous horizontal nystagmus in primary position, that is inhibited with fixation and that follows Alexander's law (the amplitude of the nystagmus increases in the gaze-direction of the primary position nystagmus fast phase) testifies for vestibular neuritis (VN). Typically, peripheral vestibular lesions have a unidirectional nystagmus that increases in the gaze direction of the fast phase (Alexander's Law) (12).

Skew deviation is a vertical ocular misalignment in primary position of gaze, and it reflects an altered otolith-ocular reflex (OOR). The physician asks the patient to fixate a central target (usually the nose of examiner), while the examiner covers the eyes of patient alternatively and observes the vertical position of the eyes. Vertical skew deviation is absent if vertigo is peripheral, while, if present, it shows a central cause (12).

If any step of HINTS indicates a central vertigo, the HINTS test is considered “central”: it implies the need for further investigation, like neuroimaging (CT or RMN), referring patients to other specialists.

### Signs of Vestibular Impairment

We searched for the signs of vestibular impairment under infra-red binocular videonystagmoscopy through:

#### Head Shaking Test

Head shaking test is considered as a useful clinical tool for detecting asymmetries between the vestibular labyrinths. The test requires that the head patient is shaked rapidly at 2 Hz oscillation for approximately 20 s in the horizontal plane. A positive test HSN was defined by the presence of at least three beats of nystagmus after stopping the head shake. These movements may cause a horizontal nystagmus where the fast phase beats toward the healthy labyrinth: this finding suggests a peripheral vestibular hypofunction, and the nystagmus has a duration that can last as long as 6 s.

Instead, the presence of a vertical or oblique nystagmus after a horizontal head shaking typically suggests pathology with a central etiology; nystagmus that is downbeating has been reported as the most common direction after horizontal head shaking in patients with migrainous vertigo (13).

#### Hyperventilation-Induced Nystagmus

Hyperventilation-induced nystagmus is commonly used because hyperventilation induces neuro-physiological modifications able to reveal latent cerebellar or vestibular diseases, while in healthy people incidence of HIN is low.

In the cases of VN and acoustic neuroma, the HIN can evoke a paretic nystagmus (in which the fast phases beat toward the healthy side) by disrupting central compensation mechanisms, but, in these pathological conditions, it can also evoke an excitatory type of nystagmus, in which the fast phases beat, on the contrary, toward the affected side. HIN is important to test in perilymphatic fistula and in the superior canal dehiscence syndrome because it can evoke either a horizontal nystagmus, in the case of larger defects in the bony wall of the semicircular canal with associated hypofunction, or torsional nystagmus, in the case of smaller defects causing a third mobile window into the inner ear (14). In cerebellar diseases, HIN can increase or evoke a downbeat nystagmus.

#### Diagnosis of Benign Paroxysmal Positional Vertigo

The benign paroxysmal positional vertigo (BPPV) is derived from a dislodged otoliths from the utricle that migrate into one of the semicircular canals (most commonly the posterior canal). BPPV is suspected when a patient reports very brief episodes of objective vertigo (generally less than 1 or 2 min), and episodes of vertigo wake up the patient from sleep (10). Clinical features essential for diagnosis are the latency, direction, time course, and duration of positional nystagmus (15).

The diagnosis is confirmed reproducing symptoms and signs using canal specific maneuvers to identify a canal-specific nystagmus. The canal-specific response is diagnosed when the head-rotation on the plane of the semicircular canal evokes a positional nystagmus. These beats in the plane of the affected canals end in the expected direction for the canal excitation or inhibition, and this positional nystagmus was studied using the Dix–Hallpike test to diagnose posterior semicircular canal BPPV (pc-BPPV) and the Pagnini-McClure maneuver to diagnose horizontal semicircular canal BPPV (hc-BPPV).

In the Dix–Hallpike maneuver, the head of patient (with sitting patient) is turned 45 degrees toward the side to be tested, and then laid back quickly into a head-hanging position. Patient refers to an attack provoked by lying down or turning over in the supine position. The canalolithiasis of posterior canal had a duration attack <1 min, the positional nystagmus is elicited after a latency of few second and the nystagmus is a combination of torsional and up-beating, and typically lasting <1 min (15).

In the Pagnini-McClure maneuver, the patient lying supine and head is elevated about 30 degrees and quickly rolled to one to another side. In this case too, vertigo is provoked by lying down or turning over in the supine position and the attack has a duration <1 min. Instead, the nystagmus is elicited after a brief latency or no latency and it beats horizontally toward the undermost ear with the head turned to either side (the nystagmus changes his direction: it is geotropic) (15).

### Statistical Analysis

Data collected were put into a database to be used for statistical analysis. Quantitative variables have been presented as mean ± SD or median (interquartile range, [IQR]), as appropriate. Categorical variables have been expressed as absolute numbers and percentages. We performed chi-squared test and one-way ANOVA to analyze the differences between demographics and different outcomes (vertigo, central nystagmus, and peripheric nystagmus). We used Fisher's exact test to examine the differences in type of nystagmus between dichotomous groups (Pfizer *vs*. all other vaccines, mRNA vaccines *vs*. others), calculating odds ratios (*OR*s) with 95% CIs. We considered results at two-tailed *p* < 0.05 as statistically significant. Data analysis was performed using R 4.1.0 (R Foundation, 2021).

## Results

Their mean age was 54.3 ± 14.1 years old, with 26 women and 7 men. We collected the general characteristics, medical history, and types of vaccines received in Table 1 and highlighted any comorbidity in Table 2. Particularly, 23 patients received Pfizer, 5 patients received Astrazeneca, 4 patients received Moderna, and 1 patient received Johnson & Johnson vaccine.

**Table 1:** General characteristics of patients (sex and age), types of vaccines received, reported symptomatology (objective, subjective vertigo, or dizziness), numbers of patients who refer associated ear, nose, and throat (ENT) symptoms.

Total number of patients	33
Men	7 (21, 21%)
Women	26 (78, 79%)
Mean age	54.53 ± 14.14
Range	24–78
**Vaccine received**	**Number of patients, (%)**
MRNA vaccine Pfizer-Biontech (Tozinameran)	23 (69, 70)
Vaccine Astrazeneca (CHADOX1 NCOV-19)	5 (15, 15)
MRNA vaccine Moderna (CX-024414)	4 (12, 12)
Vaccine Janssen (AD26.COV2.S)	1 (3, 3)
**Reported symptomatology**	
Objective vertigo	16 (48, 5)
Subjective vertigo	14 (42, 4)
Dizziness	3 (9, 1)
**Associated ENT symptoms**	
Hearing loss	4 (12, 12)
Tinnitus	6 (18, 2)
Ear fullness	2 (6, 06)
Hypersensitivity to noise	1 (3, 03)

**Table 2:** Presence of comorbidities.

**Comorbidities**	**Number of patients, (%)**
Cardiovascular	15 (45, 4)
Diabetes	4 (12, 1)
Neurologic	9 (27, 2)
Orthopedic	3 (9, 0)

*Cardiovascular: hypertension, coronaropathy and anticoagulant antiplatelet therapy; neurologic: chronic neurovascular disease, headache, and psychiatric pathologies; orthopedic: cervical or lumbar hernia*.

Symptoms included objective vertigo (16 patients, 48.5%), subjective vertigo (14 patients, 42.4%), and dizziness (3 patients, 9.1%). Of the associated ear, nose, and throat (ENT) symptoms, the most expressed was tinnitus (18.2%).

Analyzing the results of bedside examination, HINTS examination and signs of vestibular impairment, we hypothesized the probable clinical diagnosis for each patient (Table 3). In particular, 7 patients (21.2%) did not show nystagmus, 9 patients (27.3%) had and horizontal or rotatory nystagmus, 17 patients (51.5%) had a vertical or oblique nystagmus, negative HST or “central HINT.” No patient had HIN.

**Table 3:** Analysis of nystagmus and probable clinical diagnosis.

**Type of nystagmus**	**Number of patients, (%)**
No presence of nystagmus	7 (21, 21)
Presence of horizontal or rotatory nystagmus	9 (27, 27)
Presence of positive HST/ “central HINTS” or vertical or oblique nystagmus/ “central HINTS”	17 (51, 52)
**Probable clinical diagnosis**	
No presence of vestibular impairment or central etiology of vertigo/dizziness	7 (21, 21)
Benign paroxysmal positional vertigo	9 (27, 27)
Probable central etiology	17 (51, 52)

The equilibrium of a patient was evaluated with vestibulospinal stability test. Particularly, 26 patients (78.79%) presented positive Romberg Test and only 6 patients (18.18%) presented a negative Romberg Test. Moreover, 1 patient cannot execute it because of excessive instability. Of the 26 patients with positive Romberg Test, 17 patients (65.38%) presented pluridirectional oscillation, 5 patients (19.23%) presented anteroposterior oscillation, 2 patients (7.69%) presented laterolateral oscillation, and 2 patients (7.69%) showed fall tendency.

Examinating Fukuda stepping test, 21 patients (63.64%) showed a positive test and 6 patients (18.18%) showed a negative test, while 6 patients (18.18%) cannot execute it due of high instability. Particularly of this 21 patients, 10 (47.61%) showed right or left deviation, 11 (52.38%) manifested fall tendency. Only 2 patients presented frenage testing dysmetria and adiadochokinesia.

Benign paroxysmal positional vertigo was diagnosed in all patients with horizontal or rotatory nystagmus, who received a therapeutic maneuver to solve the canalolithiasis. The latter 17 cases were suggestive for vertigo of central origin, were referred to the neurologist for further clinical-instrumental investigations.

Patients with no presence of vestibular impairment or sign of central etiology of symptomatology, have been sent to other specialists, such as physiatrist or cardiologist.

We have not found any statistical difference between sex and age of patients with different outcomes (vertigo, peripheric, and central nystagmus). Restricting the analysis to patients with nystagmus (*n* = 26), we have not found any difference in the type of nystagmus comparing patients subjected to Pfizer vaccination to all the other (*OR* of having central nystagmus = 0.24, 95% *CI*: 0.004–2.65; *p* = 0.36). Similarly, patients subjected to one of the two mRNA vaccines had a non-significant *OR* = 0.42 (95% *CI*: 0.007–5.33; *p* = 0.63) of having central nystagmus.

## Discussions

The cohort included in the present study revealed the incidence of audiovestibular symptoms, in particular acute vertigo, with short onset after mRNA or adenoviral-vectored SARS-CoV-2 vaccines in patients with no history of previous COVID-19 disease.

The presence of smell and taste loss, nasal congestion, rhinorrhea, sore throat, and hearing loss has been already investigated after COVID-19 vaccination.

In a large study on 3,383 healthcare workers who received the inactivated COVID-19 vaccine (CoronaVac, Sinovac Life Sciences). Otolaryngology-specific symptoms were showed as significantly more common in subjects with a history of COVID-19 infection (16). Differently from us, in this case the authors paid attention to the previous infection and postulated that vaccination may play a triggering role in the activation of symptoms in patients with the previous COVID-19 infection.

So far, very few reports on audiovestibular symptoms after the administration of all four types of vaccine have been reported in literature. Parrino and colleagues (6) have recently described three cases of sudden unilateral tinnitus no more than 1 week later Pfizer vaccine injection in patients without previous diagnosis of COVID-19. According to the definition of Guidelines for Clinical-Safety Information on Drugs, authors reported this side effect as “very rare” (17). Indeed, it is worth citing a research letter from Formeister from Johns Hopkins University School of Medicine, Baltimore, which reported that the incidence of SSNHL occurring after COVID-19 vaccination does not exceed that of the general population, and may be lower (18). Although there is no direct evidence of the association between vaccination and SSNHL, some cases of SSNHL after COVID-19 vaccination have been recently reported (19, 20).

Many works—case series and multicentric studies—in literature during pandemic have postulated a relationship between cochleovestibular deterioration**s** and COVID-19 infection.

A recent systematic review analyzed 28 case reports/series and 28 cross-sectional studies that fit the criteria with an overall reported prevalence of 7.6% for hearing loss, 14.8% for tinnitus, and 7.2% for rotatory vertigo (21, 22).

Seventeen case reports and one case series reported hearing loss as a potential COVID-19 related symptom; of these, nine reported sensorineural hearing loss (23–34).

Although the pathophysiology of any audio-vestibular disorder linked to COVID-19 is still unknown, myriad theories have been postulated:

- cochleitis or neuritis caused by viral involvement of the inner ear or the vestibulocochlear nerve, potentially leading to vertigo, tinnitus, and hearing loss (30, 35, 36), thus a similar neurotropism could be supposed also for Coronavirus;
- immune-mediated response such as production of proinflammatory cytokines and vasculitic events that may negatively affect the audio-vestibular system (24);
- cross-reactions of antibodies or T-cells, which may misidentify inner ear antigens as the virus, leading to accidental damage to the inner ear (30);
- vascular disorders because cochlea and semicircular canals are largely susceptible to ischaemia (37, 38) due to a lack of collateral blood supply;
- endothelial dysfunction that has been suggested as a main pathophysiological process in several viral infections, such as SARS-CoV-2. The microvascular injury affects the central and peripheral nervous systems, causing a variety of neurological symptoms, such as headache and dizziness (39).

Moreover, proneness to worry and incoming stress, together with the absence of masking sounds, have been shown as potential risk factors for tinnitus worsening during pandemic (40).

We can extend the same line of reasoning to vertigo, which was the least commonly reported audio-vestibular symptom during pandemic; in many occasions (41–47), it was not clear if the findings were referring to new or pre-existing symptoms. Moreover, the majority of studies relied on self-reported questionnaires and many studies combined the prevalence of vertigo with dizziness, being the latter not necessarily of vestibular origin (41, 44, 48, 49), and mostly a common neurological manifestation of COVID-19 (50). Moreover, in 2021 was collected a case series of six patients all over the world who had sudden, severe symptoms such as vertigo, dizziness, nausea, and vomiting, with presumptive diagnosis of vestibular neuritis (51) by excluding other possible differential diagnoses.

On the contrary, large data on incidence and mechanism potentially underlying the development of ENT- and specifically cochleovestibular-effects of vaccination are still lacking.

Recently Wichova et al. (52) in a paper about otologic manifestations after COVID-19 Vaccinations reported 25 patients (83.3%) complained of hearing loss, 15 (50%) of tinnitus, 8 (26.7%) of dizziness, 5 (16.7%) of vertigo, and 9 (30%) of aural fullness. As 36.7% of the patients had a known previous underlying inner ear disorder, this work widely focused on immunologic factors that cause possible exacerbation of pre-existing otologic symptoms, due to a spike of disease specific IgG.

A randomized, cross-sectional study was performed to investigate the side effects of the BNT162b2 vaccine among healthcare workers. Vertigo-like symptoms (2.49%, 20/803), dizziness (8.34%), tinnitus (1.99%), ear pain (0.87%), changes in hearing (0.37%), and ear discharge (0.12%) were reported by the recipients (53).

According to a recent Italian cross-sectional study on 314,671 subjects vaccinated, dizziness is recorded as one of the most frequent COVID-19 vaccination adverse effects (*n*: 296, 21%) (54).

In Table 4, we present a literature review on audiovestibular disorders after COVID-19 vaccination.

**Table 4:** Literature review on audiovestibular disorders after coronavirus disease 2019 (COVID-19) vaccination.

**References**	**Vaccine platform**	**Adverse event**	**Events/Total (%)**
Parrino et al. (6)	mRNA-vaccine BNT162b2	Tinnitus	3
Tseng et al. (7)	Adenoviral-vectored vaccine: ChAdOx1 nCoV-19	Tinnitus and cochleopathy	1
Avci et al. (16)	Inactivated COVID-19 vaccine	Ear pressure	28/1,710 (1.6%)
		Dizziness	23/1,710 (1.3%)
		Hearing loss	5/1,710 (0.3%)
Formesteir et al. (18) - data from the CDC vaccine adverse events reporting system	mRNA vaccines: BNT162b2 mRNA-1273	Sudden sensorineural hearing loss	40/86,553,330 (0.3%)
Tsetsos et al. (19)	Adenoviral-vectored vaccine: ChAdOx1 nCoV-19	Sudden sensorineural hearing loss	1
Jeong and Choi (20)	Adenoviral-vectored vaccine: ChAdOx1 nCoV-19; mRNA-vaccine BNT162b2	Sudden sensorineural hearing loss	3
Wichova et al. (52)	mRNA vaccines: BNT162b2 mRNA-1273	Hearing loss	25/30 (83.3%)
		Tinnitus	15/30 (50%)
		Dizziness	8/30 (26.7%)
		Vertigo	5/30 (16.7%)
		Aural fullness	9/30 (30%)
Kadali et al. (53)	mRNA vaccine: BNT162b2	Vertigo-like symptoms	20/803 (2.49%)
		Dizziness	67/803 (8.34%)
		Tinnitus	16/803 (1.99%)
		Ear pain	7/803 (0.87%)
		Changes in hearing	3/803 (0.37%)
		Ear discharge	1/803 (0.12%)
Gianfredi et al. (54)	mRNA vaccines: BNT162b2, mRNA-1273; adenoviral-vectored vaccines: ChAdOx1 nCoV-19, Ad26.COV2.S	Dizziness	296/314,671 (21%)

As far as we know, the present study is the first clinical report about acute vertigo after COVID-19 vaccination, which describes characteristics of nystagmus and related suggested peripheral/central origin. Evoked horizontal/rotatory nystagmus was pathognomonic for BPPV and led to treatment with therapeutic maneuver. In the remaining 17 cases, peripheral vestibular dysfunction could be almost excluded if spontaneous or evoked nystagmus are absent, while vertical/oblique nystagmus and central HINT are highly suggestive for central origin disorder.

However, this work has several limitations, since it evaluates a common symptom “acute vertigo” present in different diseases with multiple pathophysiological factors. Although the HINT test demonstrates excellent sensitivity and specificity in the assessment of acute vestibular syndromes, false-positive and false-negative results do exist; all tests have been used in this study in order to reach a topodiagnosis, but a specific etiology could not be identified. Moreover, the sample size included in the study was too small and heterogeneous to establish a cause–effect relationship between acute vertigo and SARS-CoV-2 vaccines.

However, it is worth noting that all reports in literature about possible vaccination side effects have small sample sizes; this phenomenon is linked to the scarce observational time elapsed since the large-scale diffusion of vaccines, as well as the variable adherence of the population to the vaccination campaign. The most extensive data on the adverse effects have been reported by the surveillance reports drawn up by medicines agencies or were collected through online questionnaires, without ever relying on a real clinical evaluation of symptoms. This exposes to multiple and worse biases, as the reports are not clinically verified.

So far, this is the first post-vaccine clinical evaluation of the complaint “acute vertigo,” which has been investigated by ENT/otoneurological point of view, by means of nystagmus description, specific tests battery and symptoms characterization. Our results seem to demonstrate that after vaccination peripheral injuries are less frequent, which represents the contrary to what is expected in the general population. After all, these observations refer to a historical moment of particular attention to post-vaccine symptoms; it is reasonable to think that in other times patients with “acute vertigo” symptom may turn to the general practitioner, while ENT doctor is consulted mainly for vestibular disorders of peripheral origin.

The mechanism underlying the onset of acute vertigo of central origin remains unclear. SHNL after COVID-19 vaccination has been linked to an abnormal autoimmune response (mediated by circulating immune complexes or cytotoxic vestibule-cochlear autoantibodies) or a vasculitic event with subsequent localized damage to the cochlea (55).

Due to the prevalence of nystagmus of non-peripheral origin, a central nervous system involvement could be included. It is worth noting that a significant number of central and peripheral nervous system manifestations have been reported during pandemic, such as cerebrovascular disease, impaired consciousness, cranial nerve manifestations, and impaired vision (56, 57). Recent studies have unveiled neurotrophic and neuroinvasive characteristics possessed by the novel coronavirus, probably due to direct viral neurological injury or indirect neuroinflammatory and autoimmune mechanisms (57). This has ignited the search on the evidence available on the prevalence of audiovestibular symptoms among patients infected with SARS-CoV-2 (21, 22).

It is well known that mRNA vaccines against the SARS-CoV-2 virus provide human cells instructions to produce the Spike protein, thus inducing levels of anti-S and/or anti-RBD binding antibodies. A recent work as shown how spike protein subunit 1 (S1) of SARS-CoV-2 – in this case intravenously injected radio iodinated S1 (I-S1) – is capable to cross the blood–brain barrier and enter the parenchymal brain space in male mice (58). S1 is the binding protein for SARS-CoV-2; it binds to angiotensin-converting enzyme 2 (ACE2) (59) and probably other proteins as well. These mechanisms are important for understanding whether SARS-CoV-2 and S1 itself could induce responses in the brain. As ACE2 has been reported to be abundant in the brain, medulla oblongata, and temporal lobe, the hearing center becomes affected, paving the way to hearing loss.

On the other hand, an immunization anxiety-related reaction can be postulated, as anxiety has also been related to the severity and persistency of tinnitus (40, 60). It is of utmost importance to evaluate the subsequent sequelae involving not only audiovestibular system, but also connected psychological field.

Some considerations are necessary. In our cohort, the time of onset of symptoms was no longer than 48 h after vaccination. Interestingly, 9 (27.2%) patients complained of dizziness or vertigo after the first dose, while 24 (72.8%) cases had problems only after the second dose. We have to consider the frame of Immunoglobulin G (IgG) production that is at least 10–14 days after priming (61). Interesting works by Gallus et al. (62) and Dror et al. (63) about COVID-19 long-term sequelae, suggest that cochlear damage or vestibular dysfunction are mostly transitory, thus no clinically relevant impact on audiovestibular system can be found after recovery from virus. Few data about the effects of vaccination are available so far; therefore, only similarity between systems can be traced.

In conclusion, there is growing evidence from Vaccine Surveillance Reports that hearing loss, tinnitus and vertigo can be part of the clinical spectrum of COVID-19 vaccination side effects, even if available studies in literature have small sample size and do not report the difference between central or peripheral vertigo. Although, the benefits of the vaccines far outweigh the risks of possible cochleovestibular symptoms, large-scale and well-designed studies are needed to better define possible adverse reactions and long-term consequences of the COVID-19 vaccine.

In this perspective and in light of the expected third dose, our report would also be a warning to clinicians and researchers in order to point out all possible adverse events, identify possible pathophysiological mechanisms, and enlarge systematic vaccine safety studies.

## Data Availability Statement

The original contributions presented in the study are included in the article/supplementary material, further inquiries can be directed to the corresponding author/s.

## Ethics Statement

The studies involving human participants were reviewed and approved by Ethics Committee of Catania 1, G Rodolico-San Marco University Hospital. The patients/participants provided their written informed consent to participate in this study.

## Author Contributions

PD designed and carried out the study. PD, RA, DR, and PS collected data and contributed to the writing of the manuscript. PD and RA designed the plan of statistical analysis of the study. SC, IL, IT, SF, and AM revised the manuscript. All authors have critically reviewed and agreed this final version of the article.

## Conflict of Interest

The authors declare that the research was conducted in the absence of any commercial or financial relationships that could be construed as a potential conflict of interest.

## Publisher's Note

All claims expressed in this article are solely those of the authors and do not necessarily represent those of their affiliated organizations, or those of the publisher, the editors and the reviewers. Any product that may be evaluated in this article, or claim that may be made by its manufacturer, is not guaranteed or endorsed by the publisher.

## References

[1] World Health Organization (WHO) - WHO Coronavirus (COVID-19) Dashboard Available online at: https://covid19.who.int. (accessed October 06, 2021).

[2] Sadarangani M Marchant A Kollmann TR. Immunological mechanisms of vaccine-induced protection against COVID-19 in humans. Nat Rev Immunol. (2021) 21:475–84. 10.1038/s41577-021-00578-z 34211186 PMC8246128

[3] Martínez-Flores D Zepeda-Cervantes J Cruz-Reséndiz A Aguirre-Sampieri S Sampieri A Vaca L. SARS-CoV-2 vaccines based on the spike glycoprotein and implications of new viral variants. Front Immunol. (2021) 12:701501. 10.3389/fimmu.2021.701501 34322129 PMC8311925

[4] Italian Medicines Agency COVID-19 Vaccine Surveillance Report (27/12/2020-26/08/2021). Available online at: https://www.aifa.gov.it/en/farmacovigilanza-vaccini-covid-19. (accessed October 06, 2021).

[5] Chen J Cai Y Chen Y Williams AP Gao Y Zeng J. Nervous and muscular adverse events after COVID-19 vaccination: a systematic review and meta-analysis of clinical trials. Vaccines (Basel). (2021) 9:939. 10.3390/vaccines9080939 34452064 PMC8402736

[6] Parrino D Frosolini A Gallo C De Siati RD Spinato G de Filippis C. Tinnitus following COVID-19 vaccination: report of three cases. Int J Audiol. (2021) 13:1–4. 10.1080/14992027.2021.1931969 34120553

[7] Tseng PT Chen TY Sun YS Chen YW Chen JJ. The reversible tinnitus and cochleopathy followed first-dose AstraZeneca COVID-19 vaccination. QJM. (2021) 114:663–4. 10.1093/qjmed/hcab210 34297133 PMC8344939

[8] Vaccine Adverse Event Reporting System. Available online at: https://vaers.hhs.gov/. (accessed October 06, 2021).

[9] Gurley KL Edlow JA. Diagnosis of patients with acute dizziness. Emerg Med Clin North Am. (2021) 39:181–201. 10.1016/j.emc.2020.09.011 33218657

[10] Quimby AE Kwok ESH Lelli D Johns P Tse D. Usage of the HINTS exam and neuroimaging in the assessment of peripheral vertigo in the emergency department. J Otolaryngol Head Neck Surg. (2018) 47:54. 10.1186/s40463-018-0305-8 30201056 PMC6131950

[11] Kattah JC Talkad AV Wang DZ Hsieh YH Newman-Toker DE. HINTS to diagnose stroke in the acute vestibular syndrome: three-step bedside oculomotor examination more sensitive than early MRI diffusion-weighted imaging. Stroke. (2009) 40:3504–10. 10.1161/STROKEAHA.109.551234 19762709 PMC4593511

[12] Kattah JC. Use of HINTS in the acute vestibular syndrome. An overview stroke. Vasc Neurol. (2018) 3:190–6. 10.1136/svn-2018-000160 30637123 PMC6312070

[13] Zuma E Maia FC Cal R D'Albora R Carmona S Schubert MC. Head-shaking tilt suppression: a clinical test to discern central from peripheral causes of vertigo. J Neurol. (2017) 264:1264–70. 10.1007/s00415-017-8524-x 28536922

[14] Califano L Melillo MG Vassallo A Mazzone S. Hyperventilation-induced nystagmus in a large series of vestibular patients. Acta Otorhinolaryngol Ital. (2011) 31:17–26. 21808459 PMC3146331

[15] Von Brevern M Bertholon P Brandt T Fife T Imai T Nuti D. Benign paroxysmal positional vertigo: diagnostic criteria. J Vestib Res. (2015) 25:105–17. 10.3233/VES-150553 26756126

[16] Avci H Karabulut B Eken HD Faraşoglu A Çakil T Çoruk S. Otolaryngology-specific symptoms may be highly observed in patients with a history of covid-19 infection after inactivated coronavirus vaccination. Ear Nose Throat J. (2021) 8:1455613211028493. 10.1177/01455613211028493 34233498

[17] Council for International Organizations of Medical Sciences (CIOMS). (2001). Guidelines for Preparing Core Clinical-Safety Information on Drugs. Report of CIOMS Working Groups III and V (2nd ed.). Geneva: CIOMS.

[18] Formeister EJ Chien W Agrawal Y Carey JP Stewart CM Sun DQ. Preliminary analysis of association between COVID-19 vaccination and sudden hearing loss using US centers for disease control and prevention vaccine adverse events reporting system data. JAMA Otolaryngol Head Neck Surg. (2021) 147:674–6. 10.1001/jamaoto.2021.0869 34014263 PMC8138749

[19] Tsetsos N Poutoglidis A Vlachtsis K Kilmpasanis A Gougousis S. Sudden sensorineural hearing loss following the second dose of COVID-19 vaccine. Cureus. (2021) 13:e17435. 10.7759/cureus.17435 34589343 PMC8462391

[20] Jeong J Choi HS. Sudden sensorineural hearing loss after COVID-19 vaccination. Int J Infect Dis. (2021) 113:341–3. 10.1016/j.ijid.2021.10.025 34670143 PMC8520501

[21] Almufarrij I Uus K Munro KJ. Does coronavirus affect the audio-vestibular system? A rapid systematic review. Int J Audiol. (2020) 59:487–91. 10.1080/14992027.2020.1776406 32530326

[22] Almufarrij I Munro KJ. One year on: an updated systematic review of SARS-CoV-2, COVID-19 and audio-vestibular symptoms. Int J Audiol. (2021) 22:1–11. 10.1080/14992027.2021.1896793 33750252

[23] Chern A Akinrinola O. Famuyide, Gul M, Lalwani AK. Bilateral sudden sensorineural hearing loss and intralabyrinthine hemorrhage in a patient with COVID-19. Otol Neurotol. (2021) 42:e10–e14. 10.1097/MAO.0000000000002860 33301283 PMC7737860

[24] Degen C Lenarz T Willenborg K. Acute profound sensorineural hearing loss after COVID-19 pneumonia. Mayo Clin Proceedings. (2020) 95:1801–3. 10.1016/j.mayocp.2020.05.034 32753155 PMC7275185

[25] Abdel Rhman SS Abdel Wahid AA. COVID−19 and sudden sensorineuralhearing loss, a case report. Otolaryngology. (2020) 16:100198–200. 10.1016/j.xocr.2020.100198 34957357 PMC7342036

[26] Karimi-Galougahi M Naeini AS Raad N Mikaniki N Ghorbani J. Vertigo and hearing loss during the COVID-19 pandemic – is there an association? Acta Otorhinolaryngol Ital. (2020) 40:463. 10.14639/0392-100X-N0820 32519994 PMC7889249

[27] Kilic O Kalcioglu MT Cag Y Tuysuz O Pektas E Caskurlu H. Could sudden sensorineural hearing loss be the sole manifestation of COVID-19? An investigation into SARS-COV-2 in the etiology of sudden sensorineural hearing loss. Int J Infect Dis. (2020) 97:208–11. 10.1016/j.ijid.2020.06.023 32535294 PMC7289736

[28] Koumpa FS Forde CT Manjaly JG. Sudden irreversible hearing loss post COVID-19. BMJ Case Rep. (2020) 13:e238419. 10.1136/bcr-2020-238419 33051251 PMC7554505

[29] Lamounier P Franco Gonçalves V Ramos HVL Gobbo DA Teixeira RP Dos Reis PC. A 67-year-old woman with sudden hearing loss associated with SARS-CoV-2 infection. Am J Case Rep. (2020) 21:e927519. 10.12659/AJCR.927519 33139689 PMC7650213

[30] Lang B Hintze J Conlon B. Coronavirus disease 2019 and sudden sensorineural hearing loss. J Laryngol Otol. (2020) 1:1–3. 10.1017/S0022215120002145 33000716 PMC7684203

[31] Sriwijitalai W Wjwanitkit V. Hearing loss and COVID-19: a note. Am J Otolaryngol. (2020) 102473. 10.1016/j.amjoto.2020.102473 32276732 PMC7132500

[32] Fidan V. New type of corona virus induced acute otitis media in adult. Am J Otolaryngol. (2020) 41:102487. 10.1016/j.amjoto.2020.102487 32336572 PMC7161479

[33] Raad N Ghorbani J Mikaniki N Haseli S Karimi-Galougahi M. Otitis media in coronavirus disease 2019: a case series. J Laryngol Otol. (2021) 135:10–3. 10.1017/S0022215120002741 33407978 PMC7804088

[34] Mohan S Workman A Barshak M Welling DB Abdul-Aziz D. Considerations in management of acute otitis media in the COVID-19 era. Ann Otol Rhinol Laryngol. (2021) 130:520–7. 10.1177/0003489420958443 32911957

[35] Young YH. Contemporary review of the causes and differential diagnosis of sudden sensorineural hearing loss. Int J Audiol. (2020) 59:243–53. 10.1080/14992027.2019.1689432 31714154

[36] Di Nardo W Anzivino R Cattani P Santangelo R De Corso E Paludetti G. Herpes simplex virus-1 and cytomegalovirus DNAs detection in the inner ear of implanted patients with non-congenital infection. Acta Otolaryngol. (2017) 137:791–6. 10.1080/00016489.2017.1293292 28332898

[37] Chandrasekhar SS Tsai Do B Schwartz S Bontempo L Faucett E Finestone S. Clinical practice guideline: sudden hearing loss (Update). Otolaryngol-Head Neck Surg. (2019) 161:S1–s45. 10.1177/0194599819859885 31369359

[38] Cure E Cure MC. Comment on “hearing loss and COVID-19: A note”. Am J Otolaryngol Head Neck Med Surg. (2020) 41:102513. 10.1016/j.amjoto.2020.102513 32386897 PMC7192076

[39] Gavriilaki E Anyfanti P Gavriilaki M Lazaridis A Douma S Gkaliagkousi E. Endothelial dysfunction in COVID-19: lessons learned from coronaviruses. Curr Hypertens Rep. (2020) 22:63. 10.1007/s11906-020-01078-6 32852642 PMC7449866

[40] Anzivino R Sciancalepore PI Petrone P D'Elia A Petrone D Quaranta N. Tinnitus revival during COVID-19 lockdown: how to deal with it? Eur Arch Otorhinolaryngol. (2021) 278:295–6. 10.1007/s00405-020-06147-9 32572563 PMC7307941

[41] Davis HE Assaf GS McCorkell L Wei H Low RJ Re'em Y. Characterizing long COVID in an international cohort: 7 months of symptoms and their impact. EClinicalMedicine. (2021) 38:101019. 10.1016/j.eclinm.2021.101019 34308300 PMC8280690

[42] Carfi A. BernabeiR, Landi F, Gemelli Against COVID-19 Post-Acute Care Study Group. Persistent Symptoms in Patients after Acute COVID-19 JAMA. (2020) 324:603–5. 10.1001/jama.2020.12603 32644129 PMC7349096

[43] Lechien JR Chiesa-Estomba CM Place S Van Laethem Y Cabaraux P Mat Q. COVID-19 task force of YO-IFOS. Clinical and epidemiological characteristics of 1420 European patients with mild-to-moderate coronavirus disease 2019. J Intern Med. (2020) 288:335–44. 10.1111/joim.13089 32352202 PMC7267446

[44] Micarelli A Granito I Carlino P Micarelli B Alessandrini M. Self-perceived general and ear-nose-throat symptoms related to the COVID-19 outbreak: a survey study during quarantine in Italy. J Int Med Res. (2020) 48:300060520961276. 10.1177/0300060520961276 33081538 PMC8164561

[45] Munro KJ Uus K Almufarrij I Chaudhuri N Yioe V. Persistent self-reported changes in hearing and tinnitus in post-hospitalisation COVID-19 cases. Int J Audiol. (2020) 59:889–90. 10.1080/14992027.2020.1798519 32735466

[46] Iltaf S Fatima M Salman S Salam JU Abbas S. Frequency of neurological presentations of coronavirus disease in patients presenting to a tertiary care hospital during the 2019 coronavirus disease pandemic. Cureus. (2020) 12:e9846. 10.7759/cureus.9846 32953353 PMC7497771

[47] Viola P Ralli M Pisani D Malanga D Sculco D Messina L. Tinnitus and equilibrium disorders in COVID-19 patients: preliminary results. Eur Arch Otorhinolaryngol. (2021) 278:3725–30. 10.1007/s00405-020-06440-7 33095432 PMC7582442

[48] Salahuddin H Afreen E Sheikh IS Lateef S Dawod G Daboul J. Neurological predictors of clinical outcomes in hospitalized patients with COVID-19. Front Neurol. (2020) 11:585944. 10.3389/fneur.2020.585944 33193048 PMC7662675

[49] Salepci E Turk B Ozcan SN Bektas ME Aybal A Dokmetas I. Symptomatology of COVID-19 from the otorhinolaryngology perspective: a survey of 223 SARS-CoV-2 RNA-positive patients. Eur Arch Otorhinolaryngol. (2021) 278:525–35. 10.1007/s00405-020-06284-1 32794002 PMC7426010

[50] Mao L Jin H Wang M Hu Y Chen S He Q. Neurologic Manifestations of Hospitalized Patients With Coronavirus Disease 2019 in Wuhan, China. JAMA Neurol. (2020) 77:683–90. 10.1001/jamaneurol.2020.1127 32275288 PMC7149362

[51] Malayala SV Mohan G Vasireddy D Atluri P. A case series of vestibular symptoms in positive or suspected COVID-19 patients. Infez Med. (2021) 29:117–22. 33664181

[52] Wichova H Miller ME Derebery MJ. otologic manifestations after COVID-19 vaccination: the house ear clinic experience. Otol Neurotol. (2021) 42:e1213–8 10.1097/MAO.0000000000003275 34267103 PMC8443418

[53] Kadali RAK Janagama R Peruru S Malayala SV. Side effects of BNT162b2 mRNA COVID-19 vaccine: A randomized, cross-sectional study with detailed self-reported symptoms from healthcare workers. Int J Infect Dis. (2021) 106:376–81. 10.1016/j.ijid.2021.04.047 33866000 PMC8049195

[54] Gianfredi V Minerva M Casu G Capraro M Chiecca G Gaetti G. Immediate adverse events following COVID-19 immunization. A cross-sectional study of 314,664 Italian subjects. Acta Biomed. (2021) 92:e2021487. 10.23750/abm.v92iS6.12365 34739452 PMC8851022

[55] Ciorba A Corazzi V Bianchini C Aimoni C Pelucchi S Skarzyński PH. Autoimmune inner ear disease (AIED): a diagnostic challenge. Int J Immunopathol Pharmacol. (2018) 32:2058738418808680. 10.1177/2058738418808680 30376736 PMC6213300

[56] Vonck K Garrez I De Herdt V Hemelsoet D Laureys G Raedt R et al. Neurological manifestations and neuro-invasive mechanisms of the severe acute respiratory syndrome coronavirus type 2. Eur J Neurol. (2020) 27:1578–87. 10.1111/ene.14329 32416028 PMC7276727

[57] Whittaker A Anson M Harky A. Neurological Manifestations of COVID-19: a systematic review and current update. Acta Neurol Scand. (2020) 142:14–22. 10.1111/ane.13266 32412088 PMC7273036

[58] Rhea EM Logsdon AF Hansen KM Williams LM Reed MJ Baumann KK et al. The S1 protein of SARS-CoV-2 crosses the blood-brain barrier in mice. Nat Neurosci. (2021) 24:368–78. 10.1038/s41593-020-00771-8 33328624 PMC8793077

[59] Shang J Ye G Shi K Wan Y Luo C Aihara H et al. Structural basis of receptor recognition by SARS-CoV-2. Nature. (2020) 581:221–4. 10.1038/s41586-020-2179-y 32225175 PMC7328981

[60] Elarbed A Fackrell K Baguley DM Hoare DJ. Tinnitus and stress in adults: a scoping review. Int J Audiol. (2021) 60:171–82. 10.1080/14992027.2020.1827306 33000672

[61] Clem AS. Fundamentals of vaccine immunology. J Glob Infect Dis. (2011) 3:73–8. 10.4103/0974-777X.77299 21572612 PMC3068582

[62] Gallus R Melis A Rizzo D Piras A De Luca LM Tramaloni P. Audiovestibular symptoms and sequelae in COVID-19 patients. J Vestib Res. (2021) 31:381–7. 10.3233/VES-201505 33579886

[63] Dror AA Kassis-Karayanni N Oved A Daoud A Eisenbach N Mizrachi M. Auditory performance in recovered SARS-COV-2 patients. Otol Neurotol. (2021) 42:666–70. 10.1097/MAO.0000000000003037 33967243 PMC8115428

